# Cytotoxic Aaptamines from Malaysian *Aaptos aaptos*

**DOI:** 10.3390/md7010001

**Published:** 2008-12-28

**Authors:** Khozirah Shaari, Kee Cheng Ling, Zalilawati Mat Rashid, Tan Pei Jean, Faridah Abas, Salahudin Mohd. Raof, Zurina Zainal, Nordin Hj. Lajis, Habsah Mohamad, Abdul Manaf Ali

**Affiliations:** 1 Laboratory of Natural Products, Institute of Bioscience, Universiti Putra Malaysia, 43400 Serdang, Selangor, Malaysia; 2 Department of Chemical Sciences, Faculty of Science and Technology, Universiti Malaysia Terengganu, 21030 Kuala Terengganu, Terengganu, Malaysia; 3 Faculty of Biotechnology, Universiti Darul Iman, Kuala Terengganu, Terengganu, Malaysia

**Keywords:** Marine sponge, *Aaptos aaptos*, aaptaminoids, alkaloids, cytotoxicity

## Abstract

In a preliminary screen, *Aaptos aaptos* showed significant cytotoxic activity towards a panel of cell lines and was thus subjected to bioassay-guided isolation of the bioactive constituents. In addition to the known aaptamine, two new derivatives of the alkaloid were isolated from the bioactive chloroform fraction of the crude methanolic extract. Detailed analysis by NMR and mass spectroscopy enabled their identification to be 3-(phenethylamino)demethyl(oxy)aaptamine and 3-(isopentylamino)demethyl(oxy) aaptamine. The cytotoxic activities of the three alkaloids were further evaluated against CEM-SS cells.

## Introduction

Marine sponges have received a lot of research attention in the last decade and have been shown to be a prolific source of novel chemicals with promising therapeutic potentials [1–4]. The genus *Aaptos* in particular have yielded a group of 1H-benzo[d,e][1,6]-naphthyridine alkaloids, known collectively as aaptamines. *Aaptamine* [5,6] the first isolated alkaloid of this series, is a useful chemotaxonomic marker for sponges of the order Hadromerida [7] although it has also been found to occur in a sponge  of the order Haplosclerida [8]. Several members of this class of compounds have been reported to show antitumour properties [9,10], antiHIV [11], mycobacterial [11], cardiac activity [12], and more recently, sortase A inhibitory [13] and antidepressant activities [14].

In our search for pharmacologically active substances from marine organisms, we screened a number of marine sponges for cytotoxic activity against a panel of cell lines consisting of HL-60 (promyelocytic leukemia), CEM-SS (T-lymphoblastic leukemia), MCF-7 (breast cancer), HeLa (cervical cancer), HT-29 (colon cancer) and L929 (murine fibrosarcoma from mouse). In our screen, the methanolic extract of *Aaptos aaptos* showed significant cytotoxic activity towards all the cell lines. Bioassay guided fractionation of the extract led us to the isolation of two new 9*H*-benzo[d,e][1,6]-naphthyridine alkaloids, **1** and **2**, in addition to the known aaptamine (**3**). Structural elucidation of these alkaloids and their cytotoxic activity against CEM-SS cell line are described herein.

## Results and Discussion

The methanolic extract of *Aaptos aaptos* was cytotoxic against all the cell lines tested, with CD_50_ values ranging from 3.2 to 24.1 μg/ml (Table 1). Bioassay guided solvent fractionation of the crude methanolic extract of *A. aaptos* retained a maximum of activity in the chloroform soluble fraction. Chromatographic separation of the active fraction using gel filtration, normal phase chromatography and semi-preparative reverse phase HPLC, yielded small amounts of the two new aaptaminoids, **1** and **2**, in addition to aaptamine [5], **3**, as the major constituent. The structures of the two alkaloids were elucidated based on NMR and mass spectroscopic methods.

The ESI-MS (positive ion mode) of **1** exhibited [M+Na]^+^ and [M+H]^+^ pseudomolecular ion peaks at *m/z* 354.20 and 332.07, respectively. The molecular formula was determined by HR-ESIMS data (*m/z* 332.1291), to be C_20_H_17_N_3_O_2_ (calc. 332.1321). In the ^1^H NMR spectra, the signals observed at δ_H_ 8.76 (d, *J* 4.5), 7.47 (d, *J* 4.5), 6.63 (s) and 8.42 (s) were characteristic of the coupled protons H-5 and H-6, the lone proton H-7, and H-2 of the aaptamines [8], suggesting a benzo[*de*][1,6]naphthyridine skeleton. Unlike the other aaptaminoids which have been thus far reported, H-2 appeared as a singlet, thus indicating that C-3 was substituted. The ^1^H NMR spectra also exhibited a signal for only one methoxyl group, observed at δ 3.99 (s), which could be assigned to either position 8 or 9. However, the ^13^C NMR also exhibited a carbonyl signal at δ_c_ 176.3 which is consistent with the characteristic C-9 carbonyl observed in demethyl(oxy)aaptamine (**4**), previously isolated from the Okinawan *Aaptos aaptos* [6]. Careful analysis of the ^1^H-^1^H COSY, HSQC and HMBC correlations, as summarized in Table 2 further confirmed the placement of the carbonyl carbon on position 9 and substitution on C-3.

The remaining aromatic protons at δ_H_ 7.39 (t, 2H) and 7.31 (d, 3H, *J* 7.5) were those of a monosubstituted benzene ring, which must thus be part of the substituent on C-3. This was supported by LC-MS/MS^n^ experiments, where the MS/MS fragmentation of both the [M+Na]^+^ and the [M+H]^+^ ions, respectively, gave daughter ions at *m/z* 263 and 241 for the loss of 91 amu, indicative of the loss of a tropilium ion C_7_H_7_+. Another spin system, made up of a deshielded 1H broad triplet at δ 7.03, a 2H quartet at δ 3.85 and a 2H triplet at δ 3.15, was also evident from the ^1^H NMR spectra. The latter two signals were due to a pair of methylene groups, adjacent to each other and directly attached to the carbons at δ_c_ 44.3 and 35.5, respectively, based on an HSQC experiment. However, the 1H triplet at δ 7.03 was not directly attached to any carbon and based on its highly deshielded nature, the proton was deduced to be an NH proton, giving the spin system –NH-CH_2_-CH_2_- which was also evident from the ^1^H-^1^H COSY spectrum. From the COSY correlations the protons could be assigned to H-1’ (δ 7.03) correlated to H_2_-2’ (δ 3.85) which was in turn correlated to H_2_-3’ (δ 3.15). Careful analysis of the correlations observed in the HSQC and HMBC spectra connected the ethylamino spin system to the monosubstituted benzene ring. A ^3^*J* correlation from H_2_-2’ to the quaternary carbon at δ_c_ 144.1 (C-3) connected the 2-phenethylamino substituent to the main benzo[*de*][1,6]naphthyridine moiety. Based on these data, the structure of compound **1** was assigned as 3-(phenethylamino)demethyl(oxy)aaptamine.

**Figure:**
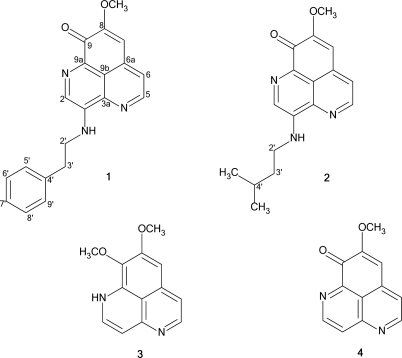

The ESI-MS (positive ion mode) of **2** exhibited an [M+Na]^+^ and [M+H]^+^ pseudomolecular ion peaks at *m/z* 320.13 and 298.20, respectively. Again, HRESIMS (*m/z* 298.1445) gave the molecular formula C_17_H_19_N_3_O_2_ (calc. 298.1477) for **2**. Meanwhile, the 1H NMR spectra of **2**, closely resembled that of **1**, showing the same C-3 substituted benzo[*de*][1,6]naphthyridine moiety, as well as the –NH-CH_2_-CH_2_- spin system of the C-3 substituent (Table 2). The difference was the absence of the phenyl protons of the C-3 substituent, which were replaced by an isopropyl group as deduced from the two equivalent methyl doublets at δ 1.05 (6H, *J* = 7.0 Hz, δ_c_ 22.6) and a methine proton at δ 1.87. The ^2^*J* and ^3^*J* HMBC correlations between H_2_-3’ and H_2_-2’, respectively, to this methine carbon (δ_c_ 26.0), assigned as C-4’, were the key correlations that connected the isopropyl group to the –NH-CH_2_-CH_2_- spin system. Again, a ^3^*J* correlation observed from H_2_-2’ to the quaternary carbon C-3 (δ_c_ 144.4) connected the isopentylamino substituent to the main benzo[*de*][1,6]naphthyridine moiety, leading us to assign **2** as 3-(isopentylamino)demethyl(oxy)aaptamine. All the three isolated alkaloids exhibited significant cytotoxic activity against CEM-SS cells with CD_50_ values of 5.3 (**1**), 6.7 (**2**) and 15.0 (aaptamine) μg/ml, respectively.

We have isolated two new aaptamines, 8-methoxy-2-(phenethylamino)-9*H-*benzo- [de][1,6]naphthyridin-9-one (**1**) and 2-(isopentylamino)-8-methoxy-9*H*-benzo[*de*][1,6]naphthyridin-9-one (**2**) from the tropical marine sponge *Aaptos aaptos*. To the best of our knowledge, this is the first report of naturally occurring C-3 substituted aaptamines. SAR studies carried out on analogs of aaptamine and isoaaptamine on several cell lines including against murine P-388 lymphocytic leukemia [9, 15–17] suggested that hydroxylation at C-9 is important for cytotoxicity, and *para*-substituted phenyl substituents on one or both of the nitrogens are important for increased activity. It is interesting to note that in this study, the cytotoxicity of the C-3 substituted aaptamines, **1** and **2**, on CEM-SS human T-lymphoblastic leukaemia cells were also observably higher than that shown by aaptamine. This suggests that C-3 substitution may also influence the cytotoxity of this class of compounds, towards this type of cell line.

## Experimental

### General Exprimental Procedures

UV and IR spectra were recorded on CARY 100 Conc UV-Vis (Varian) and Perkin-Elmer RXI FTIR spectrometers, respectively. Mass spectra were recorded on Polaris Q Mass Spectrometers (Thermo Finnigan San Jose CA), with ionization being induced by electron impact at 70eV. HRESIMS were measured using Finnigan MAT95XL-T spectrometers. LCMS/MS^n^ were performed on a ThermoFinnigan model LCQ^Deca^ (San Jose, CA). ^1^H, gCOSY, gHSQC and gHMBC NMR spectra for **1** and **2** were recorded on Varian Unity INOVA 500 Spectrometer, acquired using a gHX nanoprobe.

Adsorbent used for vacuum liquid chromatography (VLC) and column chromatography (CC) was Merck Kieselgel 60 (230–400 mesh). Gel filtrations were carried out using LH-20 (Sephadex 17-0090-01 Pharmacia Biotech). Fractions were monitored by analytical TLC, using aluminium precoated sheets (Si gel 60 F_254,_ 0.25 mm thick) with visualization under UV (254 and 366 nm), as well as with 25% H_2_SO_4_ or Dragendorff spray reagents. Analytical reversed-phase HPLC (Inertsil ODS-3 column, 7.6 x 250 mm, isocratic MeOH/H_2_O 7:3) were performed with a JASCO pump (PU-2080) equipped with a UV-Vis detector model UV-1578/1575 linked by JASCO BORWIN version 1.5 software.

### Animal material

*Aaptos aaptos* was collected from the coastal waters of Terengganu, on the eastern part of Peninsular Malaysia. A specimen (registry No. P03.015) has been deposited at the Department of Biological Science, Faculty of Science and Technology, Universiti Malaysia Terengganu.

### Extraction and isolation

Fresh samples of *Aaptos aaptos* (250 g) were cut and macerated in a high-speed blender with methanol at room temperature. The methanolic extract (5.5 g) was filtered, evaporated *in vacuo*, and lyophilized before subjected to cytotoxity assay [18] against a panel of cancer cell lines. The crude methanolic extract was further partitioned into n-hexane (1 g), chloroform (0.6 g), ethyl acetate (1.1 g)  and aqueous (2.3 g) fractions. All the fractions were assayed against CEM-SS, where the cytotoxity (CD_50_=2.4 μg/ml) was found to be concentrated in the chloroform fraction. Further isolation was thus focused on this bioactive fraction. The extract was chromatographed on a silica gel column eluting with chloroform with increasing amounts of MeOH as eluent. The fraction eluted with 20% MeOH yielded large amounts of aaptamine (50 mg). The fraction eluted with 10% MeOH was further subjected to silica gel column, eluted with dichloromethane with increasing amounts of acetone. The subfraction eluted with 30% acetone was further subjected to reversed-phase HPLC using MeOH/H_2_O 7:3 as eluent (isocratic, flow rate 3 ml/min, wavelength 366 nm) to yield 1.0 mg each of compound **1** and **2.**

Compound **1**. 3-(phenethylamino)demethyl(oxy)aaptamine or 8-methoxy-3-(phenethylamino)-9H-benzo[de][1,6]naphthyridin-9-one. Orange gum. ^1^H and ^13^C NMR data recorded in CDCl_3_ see Table 2. HRESIMS [M+H]^+^ found at m/z 332.1291, calc. 332.1321 for C_20_H_17_N_3_O_2_.

Compound **2.** 3-(isopentylamino)demethyl(oxy)aaptamine or 3-(isopentylamino)-8-methoxy-9H-benzo[de][1,6]naphthyridin-9-one. Orange gum. ^1^H and ^13^C NMR data recorded in CDCl_3_, see Table 2. HRESIMS [M+H]^+^ found at m/z 298.1445, calc. 298.1477 for C_17_H_19_N_3_O_2_.

### Evaluation of Cytotoxicity

Cytotoxic activity was measured against a panel of cell lines as described previously [18].

## Figures and Tables

**Table 1:** Cytotoxicity of crude methanolic extract and isolated compounds from *Aaptos aaptos* on cancer cell lines.

Sample	CD_50_ (μg/ml)^a^					
	HL-60	CEM-SS	MCF-7	HeLa	HT-29	L929
Crude methanolic extract	9.45 ± 0.36	5.40 ± 0.16	8.00 ± 0.08	22.80 ± 0.10	24.10 ± 0.90	3.20 ± 0.25
Compound 1	nd	5.32 ± 0.27	nd	nd	nd	nd
Compound 2	nd	6.73 ± 0.35	nd	nd	nd	nd
Aaptamine	nd	15.03 ± 0.08	nd	nd	nd	nd

a Results are expressed as IC_50_ values (μg/ml) ±SD of three experiments; nd: not determined. HL-60 (promyelocytic leukemia), CEM-SS (T-lymphoblastic leukemia), MCF-7 (breast cancer), HeLa (cervical cancer), HT-29 (colon cancer), L929 (murine fibrosarcoma from mouse).

**Table 2:** NMR data for compounds **1** and **2** recorded in CDCl_3_ (500MHz)^a^.

Position	Compound 1				Compound 2			
	δ_H_	δ_C_ (mult)	HMBC		δ_H_	δ_C_ (mult)	HMBC	
			^2^*J*	^3^*J*			^2^*J*	^3^*J*
2	8.42 s	129.8 (CH)	3	3a, 9a	8.40 s	129.8 (CH)	3	3a, 9a
3	-	144.1 (C)	-	-	-	144.4 (C)	-	-
3a	-	136.5 (C)	-	-	-	136.5 (C)	-	-
5	8.76 d (4.5)	151.0 (CH)	6	3a	8.78 d (4.5)	151.0 (CH)	6	3a
6	7.47 d (4.5)	121.8 (CH)	5	9b, 7	7.48 d (4.5)	121.8 (CH)	5	9b, 7
6a	-	nd	-	-	-	nd	-	-
7	6.63 s	106.5 (CH)	8	9, 6, 9b	6.63 s	106.3 (CH)	8	9, 6, 9b
8	-	158.1 (CH)	-	-	-	158.2 (CH)	-	-
8-OCH_3_	3.99 s	56.2 (CH_3_)	**-**	8	3.99 s	56.2 (CH_3_)	-	8
9	-	176.3 (C)	-	-	-	176.2 (C)	-	-
9a	-	134.9 (C)	-	-	8.40 s	134.6 (C)	-	-
9b	-	118.1 (C)	-	-	-	118.1 (C)	-	-
1’	7.03 bt (5.6)	-	-	-	6.93 t	-	-	-
2’	3.85 q (6.8)	44.3 (CH_2_)	3’	4’, 3	3.59 q	41.2 (CH_2_)	3’	4’, 3
3’	3.15 t (6.8)	35.5 (CH_2_)	4’, 2’	5’/9’	1.77 q	38.1 (CH_2_)	2’, 4’	5’/6’
4’	-	138.1 (C)	-	-	1.87 m	26.0 (C)	5’/6’	-
5’/9’	7.31 m	129.1* (CH)	-	3’,7’	-	-	-	-
6’/8’	7.39 dd (7.8, 7.6)	129.0* (CH)	-	4’, 5’/9’	-	-	-	-
7’	7.31m	127.2 (CH)	-	-	-	-	-	-
#CH_3_					1.05 d (7.0)	22.6 (5’, 6’) (CH_3_)	4’	3’, 5’/6’

a  *J* values are in parentheses and reported in Hz; chemical shifts are given in ppm; *,** are interchangeable; # Carbons 5’& 6’ are equivalent; nd: δ_c_ not determined since no H-C correlations could be seen in the HMBC spectrum.
